# Fatal Tracheoesophageal Puncture Leakage Associated With Lenvatinib

**DOI:** 10.7759/cureus.43490

**Published:** 2023-08-14

**Authors:** Sarah Salvatori, Tawee Tanvetyanon

**Affiliations:** 1 Medicine, University of South Florida Morsani College of Medicine, Tampa, USA; 2 Thoracic Oncology, Moffitt Cancer Center, Tampa, USA

**Keywords:** vegf chemotherapy, thyroid cancer surgery, chemotherapy-related toxicity, chemotherapy, tracheoesophageal puncture, retropharyngeal abscess, tyrosine kinase receptor inhibitors, total laryngectomy, lenvatinib

## Abstract

Tracheoesophageal puncture (TEP) is a voice restorative option adopted by many head and neck cancer patients following laryngectomy. Though generally safe, TEP may develop leakage. Lenvatinib is a tyrosine kinase inhibitor (TKI) with anti-tumoral activity against head and neck malignancies.TKIs, including lenvatinib, have been associated with organ perforation or fistula formation. There remains a paucity of literature on the association between lenvatinib and TEP leakage. In this report, we described a patient with adenoid cystic carcinoma of the larynx who had a TEP. After approximately two weeks of treatment with lenvatinib, the patient developed a leakage of TEP. Despite several interventions, the patient died three months afterward due to a retropharyngeal abscess secondary to *Fusobacterium nucleatum*. To our knowledge, this is the first report of fatal lenvatinib-associated TEP leakage. Clinicians should be cognizant of a potentially rapid development of this complication when prescribing TKI for patients with TEP.

## Introduction

Tracheoesophageal puncture (TEP) can allow for voice restoration after laryngectomy [1]. TEP represents a fistula between the posterior tracheal wall and esophagus with a one-way valve voice prosthesis to allow pulmonary air to enter the esophagus when the tracheostoma is occluded but to stay closed otherwise to prevent aspiration. The passing air helps generate an audible speech when an electrolarynx is applied. Although TEP can greatly enhance the quality of life among patients with laryngectomy, it may lead to complications such as an enlargement or leakage of the TEP, which can lead to gastric aspiration. In a meta-analysis of 27 studies, the lifetime incidence of TEP leakage varied from 1% to 29% of patients, depending on the definition of leakage [2].

Several factors are known to increase the risk of TEP leakage. These include previous radiotherapy, which can affect the elasticity of tissue [3]; gastroesophageal reflux, which can alter the mucosal function [4]; and comorbidities, such as anemia or chronic renal insufficiency, which can impair tissue integrity [5]. In addition, treatment with some tyrosine kinase inhibitors (TKI) has been proposed to be associated with TEP leakage. In 2019, Britt and Russell described a series of three patients with metastatic thyroid carcinoma who developed TEP leakage or TEP bleeding in association with TKI treatment [6]. In these cases, TEP was functioning well for a prolonged period, up until the patients were exposed to lenvatinib or vandetanib. The authors hypothesized that these TKIs may interfere with wound healing, leading to complications. In these patients, however, the complications were managed effectively by discontinuation of TKI and surgical correction.

While TEP leakage is known to be potentially serious, to our knowledge, there has been no report of fatality due to TEP leakage associated with TKI in the literature. We recently cared for a patient who had TEP for 16 years without any complications. Due to cancer progression, the patient was started on lenvatinib. After approximately two weeks of lenvatinib treatment, she began to develop symptoms from TEP leakage. Despite the discontinuation of lenvatinib, the complication persisted, and she died shortly thereafter.

## Case presentation

A 67-year-old female patient was diagnosed with adenoid cystic carcinoma of the larynx 16 years ago. She underwent laryngo-pharyngectomy and thyroidectomy with an overlying flap. The pathology report from the original surgery revealed a well-differentiated 6.0 x 2.5 x 1.5 cm adenoid cystic carcinoma with a stage of T4a N1 MX. Tracheoesophageal puncture (TEP) was also constructed. The patient received close follow-up post-surgery. She was free of disease for five years afterward, but the cancer ultimately metastasized to the lung. Over the course of a decade, she underwent several treatments, including radiation therapy for lung metastases, cytotoxic chemotherapy, and checkpoint inhibitor immunotherapy. However, the disease continued to progress. At baseline, she was not anemic and renal function was normal. She had a Karnofsky performance status of 80%. Her only symptom was occasional coughs.

**Figure 1:**
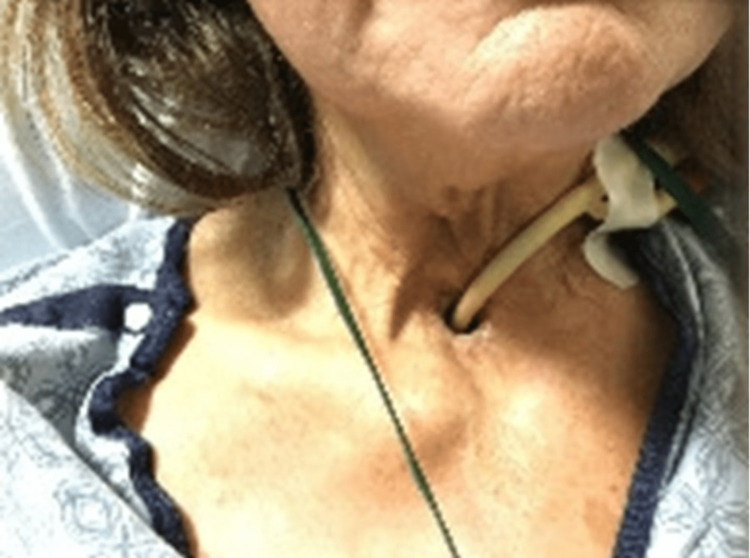
Photograph of Foley catheter through a tracheostoma

Because of her multiple previous lines of systemic therapy, the patient was found ineligible to participate in a clinical trial investigating Lenvatinib for advanced adenoid cystic carcinoma. As a result, she decided to request off-label lenvatinib for compassionate use. Prior to this, she was informed of the potential risks, and written consent was obtained. She began treatment with lenvatinib 12 mg daily orally. After treatment for 15 days, she reported an episode of severe chest pain. Computerized tomography of the chest was performed, revealing free air in the thoracic inlet. She was hospitalized. Upon inspection, her TEP puncture was enlarged to approximately 2 cm by 1 cm. A prosthesis could not be fitted. A decision was made to place a Foley catheter through the TEP into her stomach to temporarily rest her puncture site (Figure 1). Lenvatinib was discontinued.

A few weeks afterward, there was no improvement in the leakage. She subsequently underwent placement of Blom-Singer fistula prosthesis and Shiley cuffless tracheostomy. However, the patient was immediately uncomfortable after this. A decision was made to remove it, and the Foley catheter was reinstituted.

**Figure 2:**
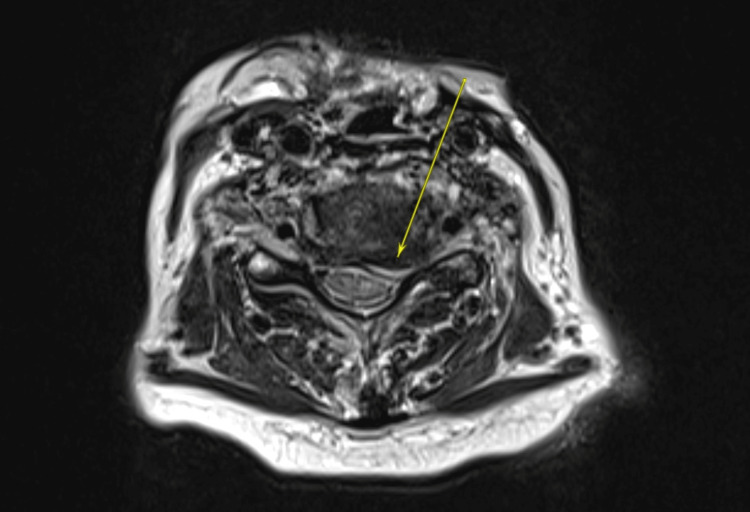
Post-contrast T2 axial MRI of the cervical spine demonstrated areas of epidural enhancement involving the ventral epidural space, including this C5-C6 level

**Figure 3:**
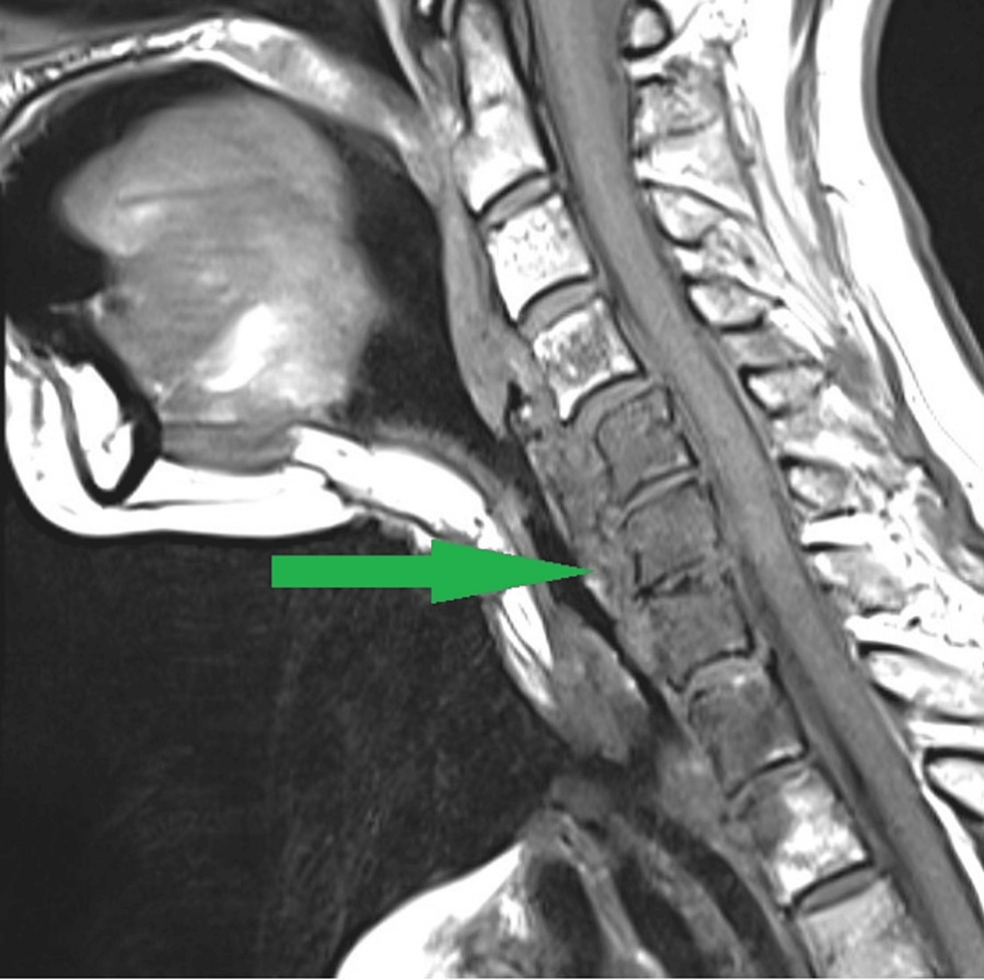
Magnetic resonance imaging of the cervical spine demonstrating an abscess in the retropharyngeal space

Throughout the next several weeks, the patient began complaining of low-grade fever and neck pain. A magnetic resonance imaging of the neck was performed, revealing a collection of gas in her retropharyngeal space at the level of C5 to C6, suggesting an abscess (Figures 2, 3). Her blood culture grew *Fusobacterium nucleatum*. Due to her declining condition, it was determined against drainage, and despite antibiotics, the patient died a few weeks afterward, approximately 12 weeks after the initiation of lenvatinib.

## Discussion

We described a patient who developed tracheoesophageal puncture (TEP) leakage after lenvatinib treatment. The patient did not have any metastasis near her stoma, and her TEP had worked well for 16 years prior to this. She did not have anemia, reflux, or other intercurrent illnesses, which could implicate the rapid development of TEP leakage. Given the close temporal relationship with lenvatinib, we believe that lenvatinib is the cause of her TEP leakage.

To our knowledge, this is the first report of lenvatinib-associated TEP leakage leading to a fatal outcome. Furthermore, this case represents the most rapid development of the complication, with an interval between lenvatinib exposure and symptomatic leakage of 15 days. The case is also unique in that it involves a patient with adenoid cystic carcinoma, a salivary gland cancer, while all previous reports are from cases with thyroid cancer.

Our literature review identified only one previously reported case of TEP enlargement associated with lenvatinib [6]. The patient developed symptoms after lenvatinib treatment for seven months. The TEP leakage was repaired with a deltopectoral flap. The same paper also described another patient who developed the complication after vandetanib treatment for 19 months. Although limited information about TEP leakage associated with tyrosine kinase inhibitor (TKI) is available in the literature, data on TKI-associated organ perforation or fistula formation may be useful for contextual comparison. For example, in a retrospective cohort of 95 patients with thyroid carcinoma treated with lenvatinib with a median dosage of 14 mg daily, there were 14 patients who ultimately developed a fistula or organ perforation during follow-ups [7]. These incidents involved bronchus, pleura, trachea, trachea-esophagus, or bladder. In this paper, the earliest complication occurred approximately one month after lenvatinib initiation.

The pathogenesis of fistula or organ perforation due to TKI is believed to be due to an inhibition of vascular endothelial growth factor (VEGF). lenvatinib is a TKI that targets VEGF receptors, along with FGF receptors, platelet-derived growth factor (PDGF) alpha, RET proto-oncogene, and KIT gene [8]. Reduced VEGF expression can lead to impaired angiogenesis and tissue formation. This can implicate the ability of the tissue to heal, which is important to maintain the integrity of TEP, a constantly dynamic structure. While discontinuation of TKI may restore VEGF function, the disruption in tissue healing may be long-lasting. There are a number of approaches to remedy the TEP leakage, including injectable soft tissue augmentation [9], adipose injection or grafting [6], or flaps [10]. However, it remains unclear which approach is best for TKI-associated TEP enlargement. In our patient, it is possible that the placement of the Blom-Singer fistula prosthesis or the use of a Foley catheter in our patient may have predisposed her to the development of a retropharyngeal abscess. Fusarium is an anaerobic organism known to cause abscesses in the head and neck region [11].

## Conclusions

As the use of tyrosine kinase inhibitors (TKIs), including lenvatinib will be increasing, our experience may be useful for clinicians when caring for similar cases in the future. It is important to consider the limitations of our observation. As a case report, we cannot establish lenvatinib as the cause of tracheoesophageal puncture (TEP) leakage. However, taken together with other reports, clinicians should be cautious when prescribing TKI for patients with TEP.
